# Case report: Aggressive NSCLC with partial BRG-1 deficiency and KRAS G12C mutation: a case study and treatment challenges

**DOI:** 10.3389/fonc.2024.1515240

**Published:** 2024-12-24

**Authors:** Chengwei Jin, Hong Ge, Dongsheng Hou, Jing Li, Mingming Tan

**Affiliations:** ^1^ Department of Cardiology, Zibo Central Hospital, Zibo, China; ^2^ Department of Respiratory and Critical Care Medicine, Zibo Central Hospital, Zibo, China; ^3^ Pathology Department, Shandong Provincial Hospital, Jinan, China; ^4^ Pathology Department, Zibo Central Hospital, Zibo, China

**Keywords:** partial BRG-1 deficiency, NSCLC, chemoimmunotherapy, KRAS G12C mutation, brain metastasis, molecular profiling

## Abstract

**Background:**

SMARCA4-deficient (BRG-1 deficient) primary thoracic tumors are rare aggressive malignancies associated with poor prognosis. While complete BRG-1 loss is well-documented, the clinical implications of partial BRG-1 deficiency remain unclear. This case report explores a case of mixed lung cancer with partial BRG-1 deficiency and KRAS G12C mutation, highlighting its clinical relevance, treatment challenges, and the importance of comprehensive molecular profiling.

**Methods:**

We performed immunohistochemistry, next-generation sequencing, and PD-L1 expression analysis to characterize the tumor. Treatment included surgical resection, chemotherapy, and immunotherapy.

**Case presentation:**

We present a case of early-stage mixed lung cancer with partial BRG-1 deficiency in a 66-year-old male, treated with surgical resection, chemotherapy, and later, a PD-1 inhibitor. Despite aggressive treatment, rapid progression to brain metastasis was observed, underscoring the need for tailored approaches.

**Conclusion:**

Partial BRG-1 deficiency may lead to aggressive clinical behavior, similar to complete BRG-1 loss. This case emphasizes the importance of comprehensive molecular profiling to guide treatment decisions and suggests further investigation into combined therapeutic strategies, including immunotherapy.

## Background

BRG-1, encoded by SMARCA4, is a key component of the SWI/SNF chromatin remodeling complex, which regulates gene expression and is involved in DNA repair, cell cycle control, and differentiation (1, 2). Complete BRG-1 loss has been linked to poor outcomes in various cancers, including lung cancer (3). However, the effects of partial BRG-1 deficiency remain underexplored. Recent studies suggest that even partial loss of the SWI/SNF complex can disrupt critical cellular pathways; leading to tumorigenesis (4).

BRG-1 deficiency has been reported in approximately 2-3% of non-small cell lung cancers (NSCLCs), with a higher prevalence in certain subtypes such as large cell carcinomas and sarcomatoid carcinomas (1). This case report explores the clinical implications of partial BRG-1 deficiency in lung cancer, highlighting the importance of comprehensive molecular profiling and the potential role of combined therapeutic strategies, including immunotherapy.

## Case presentation

### Patient overview

A 66-year-old male with a 40-year smoking history (20 cigarettes/day) was referred to our hospital following an incidental finding on a routine chest CT. The patient was asymptomatic, with no history of cough, dyspnea, or chest pain. His medical history was unremarkable except for well-controlled hypertension, and there was no family history of cancer.

On initial physical examination, the patient appeared well, and vital signs were within normal limits. Chest auscultation revealed clear breath sounds bilaterally, with no wheezes or crackles. No lymphadenopathy was detected, and the remainder of the physical examination was unremarkable.

### Diagnostic assessment

#### Imaging studies

**Figure 1:**
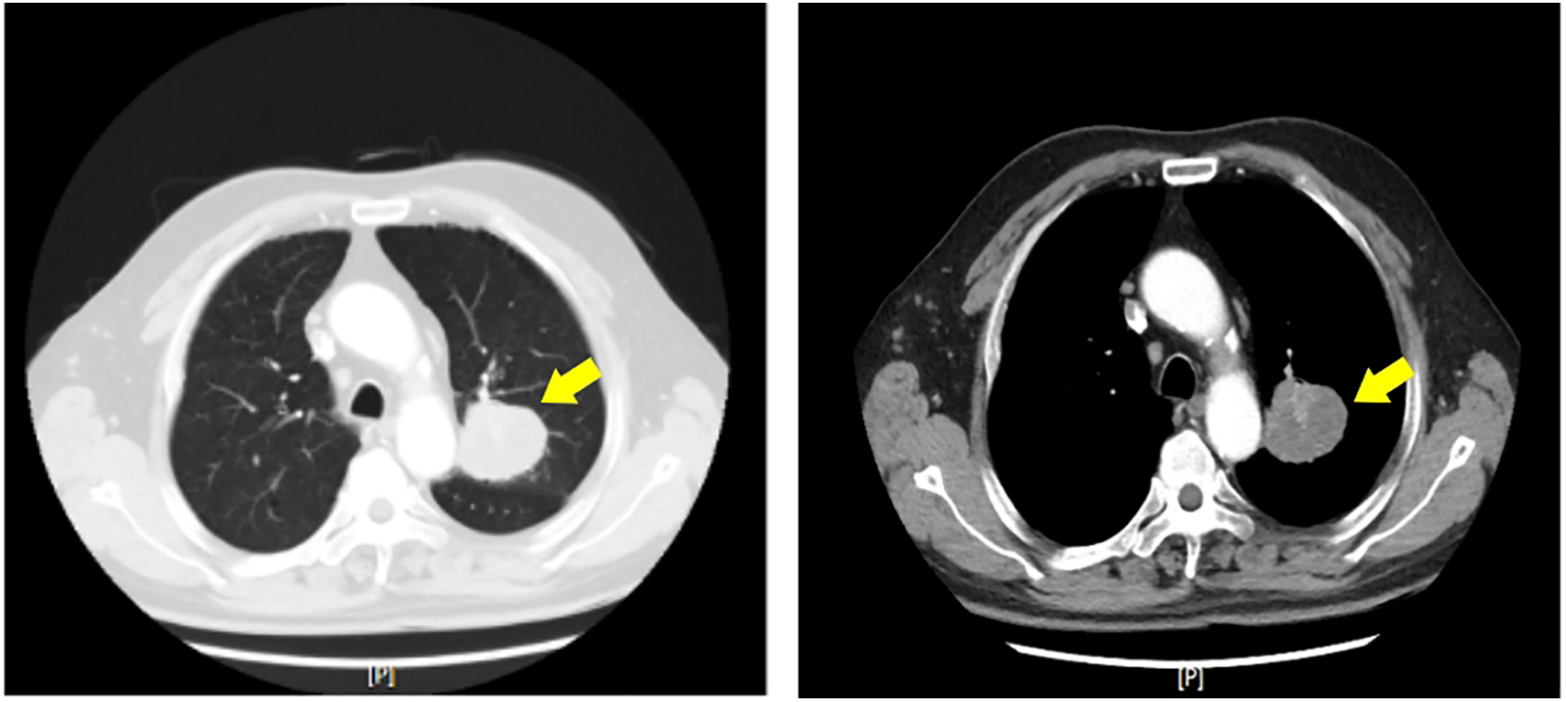
Axial computed tomography (CT) image of the chest demonstrating a solitary left upper lobe nodule (yellow arrows). The nodule exhibits spiculated margins and is surrounded by ground-glass opacity, features suggestive of malignancy.

Chest CT: A 5.2 cm * 4.8 cm solid nodule was identified in the left upper lobe, specifically in the apicoposterior segment (**Figure 1**). The nodule had spiculated margins and was surrounded by ground-glass opacity. No mediastinal lymphadenopathy or pleural effusion was observed.

PET-CT: The left upper lobe nodule showed intense FDG uptake. No other areas of abnormal uptake were noted.

**Figure 2:**
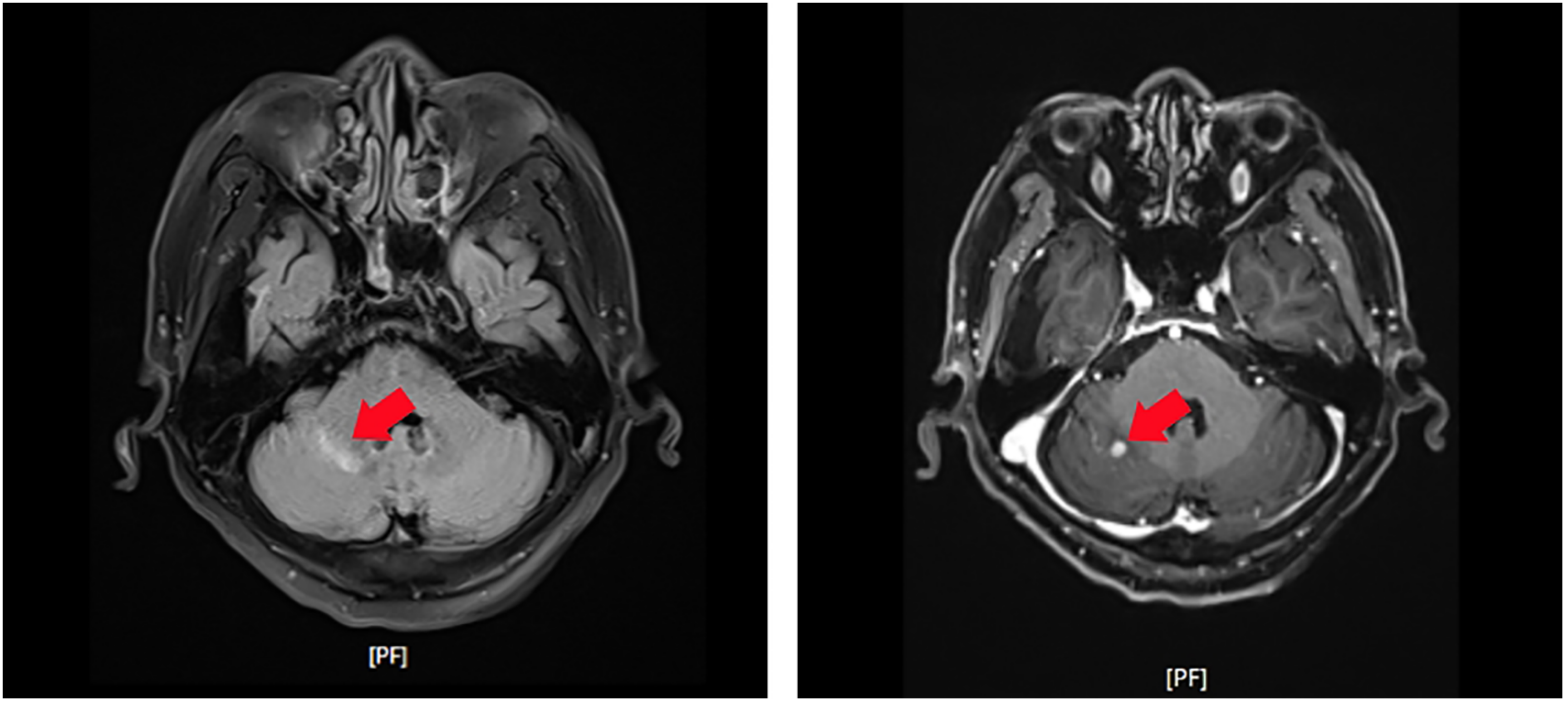
Contrast-enhanced T1-weighted magnetic resonance imaging (MRI) of the brain revealing a small enhancing lesion in the right cerebellar hemisphere (red arrows). The lesion’s characteristics are consistent with metastatic disease.

Brain MRI (post-operative): A 0.6 cm * 0.5 cm enhancing lesion was detected in the right cerebellar hemisphere, consistent with metastasis (**Figure 2**).

### Laboratory tests

#### Molecular testing methods

We assessed BRG-1 deficiency using immunohistochemistry (IHC). IHC was performed using the anti-SMARCA4 antibody (clone EPNCIR111A, Abcam) on formalin-fixed, paraffin-embedded tissue sections. BRG-1 expression was quantified by assessing the percentage of tumor cells with positive nuclear staining. Partial deficiency was defined as 20-80% of tumor cells showing loss of BRG-1 expression.

PD-L1 expression was evaluated using the Dako 22C3 pharmDx assay.

KRAS mutation was detected using PCR-based assays and confirmed by NGS. We conducted NGS using the Ion Torrent platform with a custom panel covering 50 cancer-related genes, including SMARCA4 and KRAS.

#### Pathological findings

**Figure 3:**
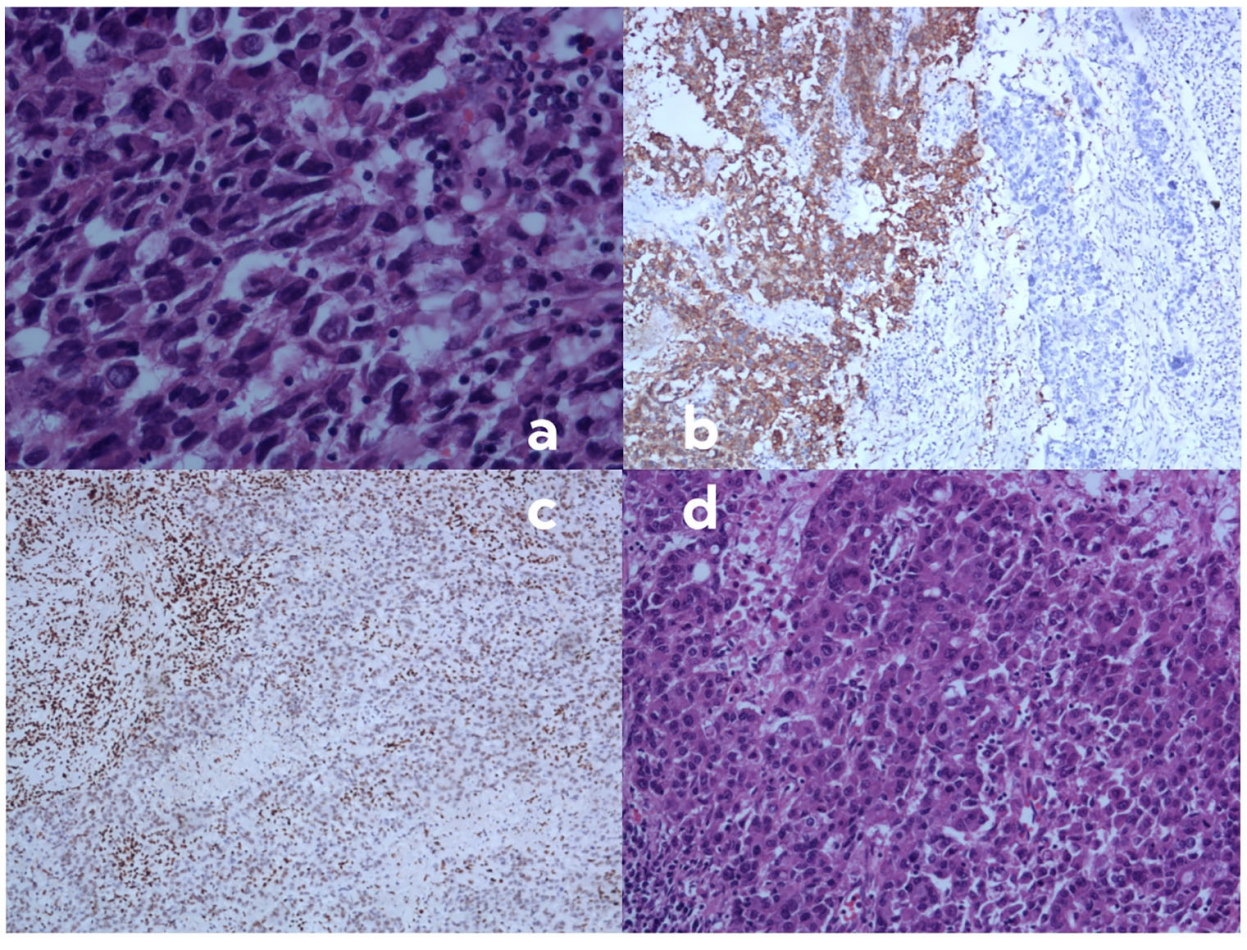
Histopathological and immunohistochemical findings of the resected tumor specimen. **(A)** Hematoxylin and eosin (H&E) staining demonstrating highly atypical cells with eosinophilic cytoplasm, increased nuclear-to-cytoplasmic ratio, poor cellular cohesion, and sarcomatoid morphology. These cells exhibit focal positivity for synaptophysin (SYN) and chromogranin A (CGA), with diminished or absent BRG1 expression (magnification ×400; scale bar, 50 μm). **(B)** Immunohistochemical staining for synaptophysin (SYN) showing a heterogeneous pattern. The right region of the image displays tumor cells negative for SYN, while the left region shows SYN-positive tumor cells, indicating neuroendocrine differentiation in a subset of the tumor. **(C)** Immunohistochemical staining for BRG1 revealing loss of expression in tumor cells. Note the positive internal control in stromal lymphocytes, confirming the validity of the staining procedure. **(D)** High-power view of H&E staining (magnification ×400; scale bar, 50 μm) showing the solid, sheet-like, and nested infiltrative growth pattern of the tumor. Cells are medium to large in size with prominent nucleoli, characteristic of poorly differentiated non-small cell lung carcinoma.

Histopathological examination revealed a mixed poorly differentiated non-small cell lung cancer with neuroendocrine features. The tumor showed areas of solid growth with focal glandular differentiation. Tumor cells exhibited high nuclear-to-cytoplasmic ratios, prominent nucleoli, and frequent mitoses (>10 per 2 mm2). Focal areas of necrosis were present. Immunohistochemistry showed partial loss of BRG-1 expression, with approximately 40% of tumor cells retaining some degree of nuclear staining (**Figure 3**).

Clinical Staging:

Based on the 8th edition of the TNM classification for lung cancer (5), the initial clinical stage was determined to be (stage IIB).

### Patient clinical timeline

#### Rationale for treatment decisions

We chose surgical resection as the initial treatment due to the early-stage presentation (clinical stage IIB) and the patient’s good performance status (**Table 1**). Following surgery, we administered adjuvant chemotherapy based on the poorly differentiated histology and the presence of neuroendocrine features, (**Table 2**). We selected etoposide and cisplatin due to their efficacy in both NSCLC and neuroendocrine tumors (**Table 1**).

**Table 1:** Patient treatment timeline and clinical course.

Date	Event
**May 6, 2024**	Diagnosis confirmed; video-assisted thoracoscopic surgery (VATS) left upper lobectomy with mediastinal lymph node dissection performed
**May 13, 2024**	Pathology report confirmed diagnosis of mixed poorly differentiated NSCLC with neuroendocrine features (pT2aN0M0)
**June 26 - July 18, 2024**	Adjuvant chemotherapy: Two cycles of etoposide (100 mg/m2, days 1-5) + cisplatin (75 mg/m2, day 1-3) every 21 days
**August 7, 2024**	Brain MRI detected 1.5 cm metastasis in right cerebellar hemisphere; gamma knife radiosurgery performed (margin dose 18 Gy)
**August 25, September 14, October 06, October 27, 2024**	Immunotherapy initiated: carrelizumab (200 mg every 3 weeks) + paclitaxel (175 mg/m2) + carboplatin (AUC 5) every 3 weeks
**September 27, 2024**	CT showed stable disease in chest; Brain MRI showed decreased size of cerebellar metastasis
**November 19, 2024**	CT showed stable disease in chest; Brain MRI shows that the original tumor lesions have disappeared compared to before.

**Table 2:** Laboratory and molecular testing results at initial diagnosis.

Marker	Result	Normal Range
**Neuron-Specific Enolase**	16.80 ng/ml (0-16.3 ng/ml)	0-16.3 ng/ml
**Carcinoembryonic Antigen**	5.09 ng/ml (0-5.0 ng/ml)	0-5.0 ng/ml
**CKAE1/AE3**	Positive	N/A
**Vimentin**	Negative	N/A
**BRG1**	Partial deficiency	N/A
**Ki-67**	70%	N/A
**PD-L1 protein immunohistochemistry**	TPS=5%, CPS=20	N/A
**KRAS Mutation**	G12C mutation	N/A
**TTF-1**	Positive	N/A
**Synaptophysin**	Focally positive	N/A
**Chromogranin A**	Negative	N/A

Upon discovery of brain metastasis, we initiated immunotherapy combined with chemotherapy, considering PD-L1 protein immunohistochemistry: TPS=5%, CPS=20, (**Table 2**) and emerging data on the efficacy of combined chemoimmunotherapy in advanced NSCLC (5). Carrelizumab was selected as the PD-1 inhibitor due to its demonstrated efficacy in NSCLC and its availability in our region (**Table 1**). The addition of paclitaxel and carboplatin to the regimen was based on the synergistic effects observed with combined chemoimmunotherapy in recent clinical trials (**Table 1**).

**Figure 4:**
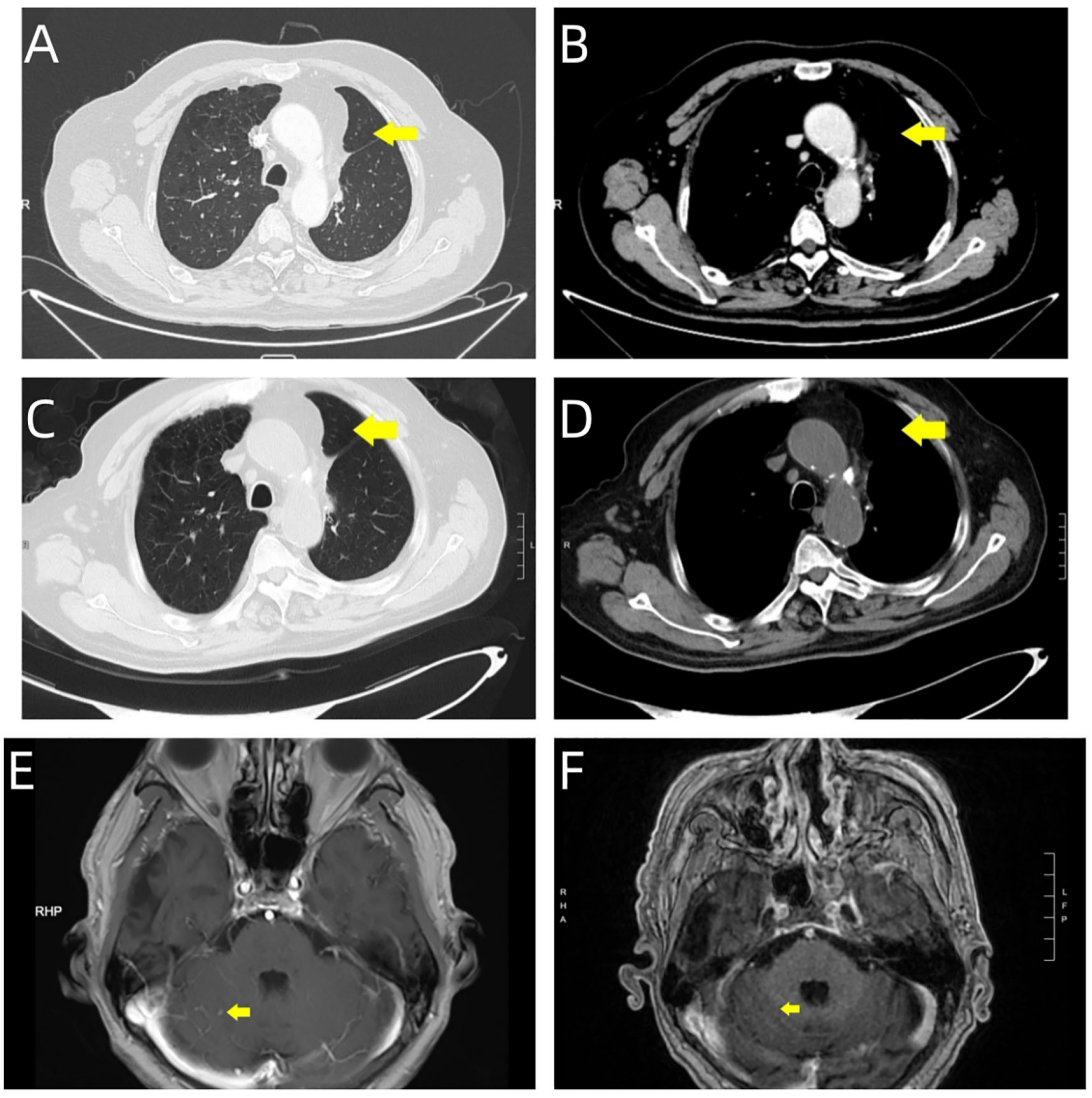
Serial imaging evaluation during follow-up period. Post-surgical surveillance imaging at two time points demonstrates treatment response: September 27, 2024. **(A, B)** Chest CT reveals post-surgical changes in the left lung, characterized by linear high-density opacities within the operative field (yellow arrows). **(E)** Brain MRI shows marked reduction in the right cerebellar hemispheric metastatic lesion (yellow arrow). November 19, 2024. **(C, D)** Follow-up chest CT demonstrates stable post-surgical changes, with persistent linear high-density opacities in the operative field (yellow arrows). **(F)** Brain MRI confirms complete resolution of the right cerebellar hemispheric lesion, with normalization of signal intensity at the prior metastatic site (yellow arrow).

The patient demonstrated excellent initial tolerability to the therapeutic regimen, experiencing only mild adverse effects (Grade 1 fatigue and nausea according to CTCAE criteria). The drug demonstrated remarkable efficacy: follow-up chest CT showed only post-surgical changes, while brain MRI revealed complete resolution of cerebellar metastases (**Figure 4**). Sequential quality of life evaluations utilizing the EORTC QLQ-C30 assessment tool revealed sustained baseline functional scores, with notable improvements in emotional and social functioning domains following immunotherapy initiation. During the most recent clinical assessment, however, the patient developed immune-mediated adrenocortical insufficiency, presenting with peripheral muscle weakness and decreased appetite. Prompt initiation of systemic glucocorticoid therapy resulted in successful resolution of these symptoms.

## Discussion

This case highlights several key aspects in treating BRG-1-deficient lung cancer: the impact of partial BRG-1 deletion, the potential role of immunotherapy, the effects of concurrent KRAS G12C mutation, and the importance of comprehensive molecular analysis.

Mechanistically, BRG-1 is crucial for maintaining chromatin structure and gene expression (6). Partial deletion can still disrupt key pathways, leading to uncontrolled cell growth. Romero et al. demonstrated that BRG1 acts as a tumor suppressor by antagonizing Myc activity and promoting cell differentiation (7). Even partial loss of BRG1 function may impair these tumor-suppressive effects, resulting in a more aggressive cancer phenotype. In this case, despite initial surgical resection and adjuvant chemotherapy, the tumor rapidly progressed to brain metastases, indicating high invasiveness. The case exhibited similar aggressive characteristics to documented cases of complete BRG-1 deficiency, suggesting that even partial deletion significantly alters treatment response.

While there is no standardized treatment protocol for BRG-1-deficient lung cancer, numerous preliminary clinical studies have provided new opportunities for treating these tumors, including platinum-based chemotherapy drugs and immune checkpoint inhibitors (ICIs). Among these, immunotherapy combined with chemotherapy is the most favored approach (8, 9). According to a case reported by Yoshida et al., using atezolizumab combined with bevacizumab, paclitaxel, and carboplatin as first-line treatment can provide up to 17 months of progression-free survival (PFS), whereas overall survival (OS) was only a few months without combined immunotherapy (10). Roberts et al. found that BRG-1 deficiency may alter the tumor microenvironment, potentially increasing sensitivity to immunotherapy (6). Studies have demonstrated that NSCLC patients with BRG-1 deficiency have a higher proportion of PD-L1 positive cases and significantly elevated Tumor Mutational Burden (TMB) levels (11, 12). Additionally, high TMB suggests that immune checkpoint inhibitors might be an attractive consideration for BRG-1-deficient thoracic sarcomatoid tumors, working synergistically with enhanced immunotherapy responsiveness in SWI/SNF-altered tumors (13). In a classic case mentioned, a 43-year-old male smoker with SMARCA4-dNSCLC developed multiple pulmonary metastases and recurrence after standard chemotherapy. Whole-exome sequencing showed PD-L1 loss of expression but high TMB, and fourth-line nivolumab treatment achieved disease control for over 14 months (14). In this case, the patient received combined immunotherapy with carrelizumab, achieving good efficacy, not only reaffirming that immune inhibitors can effectively treat poorly differentiated lung cancer with partial BRG-1 deficiency but also providing a new option that is more economical and accessible for Chinese patients.

As a chromatin remodeling factor, SWI/SNF complex dysfunction may alter the expression profile of immune-related genes. For instance, PBRM1, a crucial component of the SWI/SNF complex, has been extensively studied for its impact on immunotherapy sensitivity when deficient. Multiple studies confirm that PBRM1 deficiency can enhance sensitivity to immune checkpoint inhibitors by altering the tumor immune microenvironment and enhancing immune-related gene expression. Pan et al. found that PBRM1 deficiency enhances response to PD-1 inhibitors through enhanced interferon signaling pathways, validated in renal cell carcinoma (15). Miao et al. reported higher response rates to immune checkpoint inhibitors and significantly prolonged overall survival in renal cell carcinoma patients with PBRM1 mutations, suggesting PBRM1 as a predictive marker for immunotherapy efficacy (16). Although this research focused on different cancer types and SWI/SNF components, it indicates potential links between SWI/SNF complex deficiencies and immunotherapy response, providing important references for understanding the role of other SWI/SNF complex components like BRG-1 in immunotherapy. The present case showed good response to immunotherapy, suggesting BRG-1 deficiency may affect treatment sensitivity through similar mechanisms.

Genomic analyses have demonstrated that BRG1 mutations are present in approximately 10% of non-small cell lung cancer (NSCLC) cases. These genetic alterations commonly occur in conjunction with mutations in other critical tumor-associated genes, including STK11, KEAP1, TP53, and KRAS, indicating intricate molecular interplay in oncogenesis (12). The presence of KRAS G12C mutations may exacerbate the aggressiveness of tumors. The presence of KRAS G12C mutation may have exacerbated tumor invasiveness. KRAS, KEAP1, and STK11 are classic smoking-related NSCC mutations (1, 10, 17), and existing data indicate that SMARCA4-dNSCLC primarily occurs in male smokers. This suggests smoking may be one factor contributing to co-mutation of BRG1 deficiency and KRAS. Romero et al.’s research indicates that BRG-1 deficiency can significantly impact the progression of KRAS-dependent tumors, suggesting Brg1 is a determinant in context-dependent Kras-driven pancreatic tumor development (18). However, it’s noteworthy that in SMARCB1-deficient undifferentiated pancreatic carcinoma, KRAS is typically wild-type, as reported in a recent study (19). This study also mentioned the therapeutic significance of innovative development strategies targeting the SWI/SNF complex’s genomic foundation, including EZH2 inhibition (NCT03213665), SMARCA2 degraders (NCT05639751), or immunotherapy. Januario et al. demonstrated that SWI/SNF-mutant cancers (including those with SMARCA4/BRG1 mutations) typically show increased sensitivity to EZH2 inhibitors (20), suggesting that the combination of BRG-1 deficiency and KRAS mutation may not only alter signaling pathways and immunological characteristics but also create specific vulnerabilities that can be therapeutically exploited.

The potential synergy between EZH2 inhibitors, KRAS inhibitors, and immunotherapy in cases of partial BRG-1 deficiency and KRAS mutation warrants further investigation. Ongoing clinical trials are exploring these combinations in molecularly defined subgroups of NSCLC patients (21–23).

## Conclusion

This case represents one of the few documented instances of partial BRG-1 deficiency in NSCLC, providing valuable insights into its clinical behavior. It emphasizes the importance of comprehensive molecular analysis in guiding treatment decisions. BRG-1 deficiencies are more common in poorly differentiated cancers, such as sarcomatoid carcinomas, often accompanied by neuroendocrine differentiation (10, 24, 25). However, routine pathological examination typically relies on small tissue samples obtained through lung biopsy or bronchoscopy, which may overlook BRG-1 testing due to limited tissue samples, potentially leading to false negatives and explaining poor clinical outcomes in some cases. This case of partial BRG-1 deficiency in lung cancer demonstrates that even incomplete BRG-1 loss can lead to aggressive tumor behavior and resistance to conventional treatments. Long-term follow-up studies and further validation in a larger population of patients with partial BRG-1 deficiency are needed to better understand the natural history and treatment outcomes of these patients. The identification of partial BRG-1 deficiency, KRAS mutations, and PD-L1 expression all contribute to treatment strategy development. Comprehensive molecular analysis, including assessment of BRG-1 status, KRAS mutations, and PD-L1 expression, is crucial for developing personalized treatment approaches. As our understanding of these molecular alterations deepens, we may be able to develop more targeted and effective treatment strategies. It is worth exploring the possibility of combining targeted therapies (such as KRAS G12C inhibitors, EZH2 inhibitors) with immunotherapy for patients with BRG-1 deficiency and KRAS mutations. Such combinations may help overcome resistance mechanisms and improve treatment outcomes for this unique patient population.

## Data Availability

The original contributions presented in the study are included in the article/supplementary material. Further inquiries can be directed to the corresponding author.
